# Correction: Mansoor et al. Effect of Currently Available Nanoparticle Synthesis Routes on Their Biocompatibility with Fibroblast Cell Lines. *Molecules* 2022, *27*, 6972

**DOI:** 10.3390/molecules28104173

**Published:** 2023-05-18

**Authors:** Afsheen Mansoor, Zohaib Khurshid, Emaan Mansoor, Muhammad Talal Khan, Jithendra Ratnayake, Asif Jamal

**Affiliations:** 1 Department of Dental Material Sciences, School of Dentistry, Shaheed Zulfiqar Ali Bhutto Medical University, Islamabad 45320, Pakistan; 2 Department of Prosthodontics and Dental Implantology, College of Dentistry King Faisal University, Al-Ahsa 31982, Saudi Arabia; 3 Islamic International Dental College, Riphah International University, Islamabad 6000, Pakistan; 4 Department of Dental Biomaterials, Bakhtawar Amin Medical and Dental Collage, Multan 60650, Pakistan; 5 Department of Oral Sciences, Faculty of Dentistry, University of Otago, Dunedin 9016, New Zealand; 6 Department of Microbiology, Quaid-i-Azam University, Islamabad 45320, Pakistan

**Error in Figure**

In the original publication [1], there was a mistake in Figure 15 as published. Due to the extensive number of data included in this study, the images in Figure 15a–t were uploaded erroneously and contained repeated images. The corrected Figure 15 shows below. 

The authors state that the scientific conclusions are not affected by this correction. This correction was approved by the Academic Editor. The original publication has also been updated. 

## Figures and Tables

**Figure 15:**
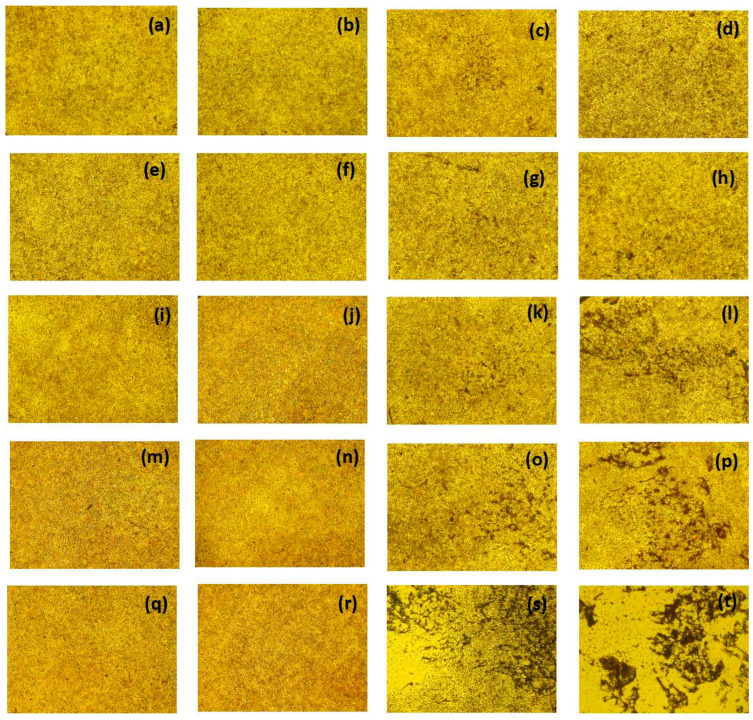
Mouse fibroblast’s cell morphology exposed to control group prepared by water on first day, 15th day, 31st day, 41st day and 51st day, showing normally large, elongated flat cells with cytoplasm (**a**,**e**,**i**,**m**,**q**). Mouse fibroblast’s cell morphology exposed to experimental group of titanium nanoparticles prepared by *Bacillus Subtilus* on first day, 15th day, 31st day, 41st day and 51st day showing normally large, elongated flat cells with cytoplasm (**b**,**f**,**j**,**n**,**r**). Mouse fibroblast’s cell morphology exposed to experimental group of titanium nanoparticles prepared by *Cassia fistula* on first day, 15th day, 31st day, 41st day and 51st day, showing initiation of pore formation (**c**), increased pore formation (**g**), increased pore formation and mild degradation (**k**), increased pore formation and mild degradation (**o**) and loss of normal spindle shape (**s**). Mouse fibroblast’s cell morphology exposed to experimental group of titanium nanoparticles prepared by hydrothermal heating on the first day, 15th day, 31st day, 41st day and 51st day, showing slight degradation (**d**), increased pore formation and degradation (**h**), greater disruption (**l**), complete loss of cell symmetry (**p**) and entire loss of normal size, shape and symmetry of cell (**t**).
